# Advancing behavioral interventions for African American/Black and Latino persons living with HIV using a new conceptual model that integrates critical race theory, harm reduction, and self-determination theory: a qualitative exploratory study

**DOI:** 10.1186/s12939-022-01699-0

**Published:** 2022-07-16

**Authors:** Marya Gwadz, Sabrina R. Cluesman, Robert Freeman, Linda M. Collins, Caroline Dorsen, Robert L. Hawkins, Charles M. Cleland, Leo Wilton, Amanda S. Ritchie, Karen Torbjornsen, Noelle R. Leonard, Belkis Y. Martinez, Elizabeth Silverman, Khadija Israel, Alexandra Kutnick

**Affiliations:** 1 Intervention Innovations Team Lab (IIT-Lab), New York University Silver School of Social Work, New York, NY USA; 2 Center for Drug Use and HIV Research, School of Global Public Health, New York University, New York, NY USA; 3 Independent Consultant, Brooklyn, NY USA; 4 Department of Social and Behavioral Sciences, School of Global Public Health, New York University, New York, NY USA; 5 Rutgers University School of Nursing, Newark, NJ USA; 6 Division of Biostatistics, Department of Population Health, New York University School of Medicine, New York, NY USA; 7 Department of Human Development, State University of New York at Binghamton, Binghamton, NY USA; 8 Faculty of Humanities, University of Johannesburg, Johannesburg, South Africa; 9 School of Global Public Health, New York University, New York, NY USA; 10 Department of Psychology, Fordham University, Bronx, NY USA

**Keywords:** Qualitative, Critical race theory, Harm reduction, Self-determination theory, HIV care continuum, Structural racism, Racial, Ethnic inequalities, Intervention, Motivational interviewing

## Abstract

**Background:**

Rates of participation in HIV care, medication uptake, and viral suppression are improving among persons living with HIV (PLWH) in the United States. Yet, disparities among African American/Black and Latino PLWH are persistent, signaling the need for new conceptual approaches. To address gaps in services and research (e.g., insufficient attention to structural/systemic factors, inadequate harm reduction services and autonomy support) and improve behavioral interventions, we integrated critical race theory, harm reduction, and self-determination theory into a new conceptual model, then used the model to develop a set of six intervention components which were tested in a larger study. The present qualitative study explores participants’ perspectives on the study’s acceptability, feasibility, and impact, and the conceptual model’s contribution to these experiences.

**Methods:**

Participants in the larger study were African American/Black and Latino PLWH poorly engaged in HIV care and with non-suppressed HIV viral load in New York City (*N* = 512). We randomly selected *N* = 46 for in-depth semi-structured interviews on their experiences with and perspectives on the study. Interviews were audio-recorded and professionally transcribed verbatim, and data were analyzed using directed qualitative content analysis.

**Results:**

On average, participants were 49 years old (SD = 9) and had lived with HIV for 19 years (SD = 7). Most were male (78%) and African American/Black (76%). All had taken HIV medication previously. Challenging life contexts were the norm, including poverty, poor quality/unstable housing, trauma histories exacerbated by current trauma, health comorbidities, and substance use. Participants found the study highly acceptable. We organized results into four themes focused on participants’ experiences of: 1) being understood as a whole person and in their structural/systemic context; 2) trustworthiness and trust; 3) opportunities for self-reflection; and 4) support of personal autonomy. The salience of nonjudgment was prominent in each theme. Themes reflected grounding in the conceptual model. Participants reported these characteristics were lacking in HIV care settings.

**Conclusions:**

The new conceptual model emphasizes the salience of systemic/structural and social factors that drive health behavior and the resultant interventions foster trust, self-reflection, engagement, and behavior change. The model has potential to enhance intervention acceptability, feasibility, and effectiveness with African American/Black and Latino PLWH.

**Supplementary Information:**

The online version contains supplementary material available at 10.1186/s12939-022-01699-0.

## Background

Racial/ethnic disparities in engagement in HIV care, HIV medication uptake, medication adherence, and HIV viral suppression are serious and persistent in the United States [1]. These disparities are a grave public health concern, because high rates of consistent involvement along this HIV care continuum are needed to enable persons living with HIV (PLWH) to achieve optimal health and wellbeing and ultimately end the HIV epidemic [1]. Although engagement rates have improved among PLWH as a whole in the past decade in the United States [2], a substantial proportion of PLWH, mainly those from African American/Black and Latino (AABL) racial/ethnic backgrounds, are poorly engaged or inconsistently engaged and, in fact, often do not sustain HIV viral suppression [2]. For example, current national data indicate that while 67% of White PLWH receive HIV primary care, only 58–59% of AABL-PLWH do so, and while 57% of White PLWH are HIV virally suppressed, only 48% of Latino and 43% of African American/Black PLWH achieve this important health indicator [2]. Of further concern, rates of sustained HIV viral suppression are lowest among African American/Black PLWH: an estimated 41% sustain viral suppression, compared to 50% among Latino and 56% among White PLWH [3]. Long periods of non-suppressed HIV viral load have potential serious adverse effects on health; they can damage immune system functioning and reduce quality of life [4]. Moreover, those with non-suppressed HIV viral load have elevated chances of forward transmission of HIV to others compared to those with sustained viral suppression [5]. The United States public health system has set a goal of ending the HIV epidemic by 2030 [6]. This will entail 95% of those living with HIV being diagnosed, 95% of those diagnosed receiving HIV medication, and 95% of all people receiving medication achieving HIV viral suppression, called “95–95-95 goals” [7]. However, the 95–95-95 goals will not be achieved without reducing or eliminating racial/ethnic inequities along the HIV care continuum [8]. These persistent inequities signal the need for new conceptual models and approaches to improve research, interventions, and HIV clinical care for those with the greatest barriers to consistent engagement along the HIV care continuum – AABL-PLWH.

**Fig. 1:**
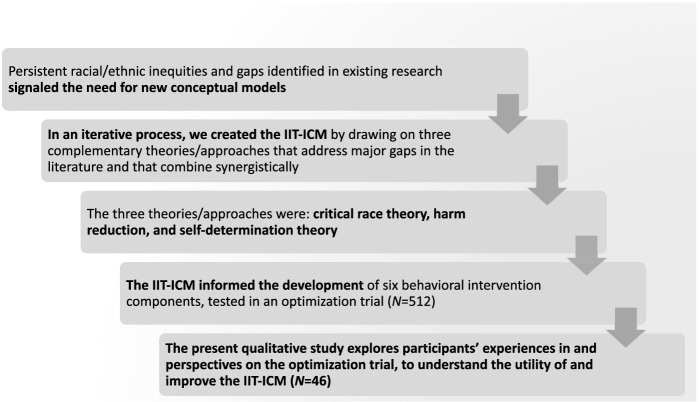
Schematic describing the steps leading to the present study

The overall goal of the present study is to evaluate the acceptability, feasibility, and effects of a behavioral intervention research study grounded in a new conceptual model developed by our research team. The need for this new model surfaced in response to a number of concerns in the HIV field. First, a substantial proportion of PLWH are out-of-care and not taking HIV medications, mainly AABL-PLWH, as described above. This subpopulation of AABL-PLWH is more challenging to involve in research than their peers who are well-engaged along the HIV care continuum and thus they are under-studied in research [9–12]. Indeed, they have serious barriers to engagement along the continuum, and these challenges are also relevant for (and complicate) behavioral intervention delivery. Moreover, improvements in HIV medication regimens have created a large population of long-term HIV survivors, including AABL-PLWH, and for these individuals, their HIV management has periods of stability and times of disruption [9, 13–15]. Effective behavioral interventions must be tailored to the population of interest, and clearly interventions are needed designed specifically for those with the greatest barriers to engagement along the care continuum, including AABL-PLWH long-term survivors who are often absent from HIV care and not taking HIV medications. The present study focuses on AABL-PLWH poorly engaged in HIV care and with non-suppressed HIV viral load. Second, it is well-established that the effects of behavioral interventions to improve HIV medication adherence to support HIV viral suppression have only modest effects which wear off when the intervention ceases [16, 17]. Clearly efficient interventions with durable effects are needed. Last, despite major investments in public health programs and research to reduce racial/ethnic inequities along the HIV care continuum, rates of engagement along the continuum have remained unacceptably low among AABL-PLWH for decades [2]. These concerns about the set of interventions and services currently available to AABL-PLWH, taken together, signal the need for new conceptual and treatment approaches. In the sections that follow, we review the main barriers that AABL-PLWH experience to the HIV care continuum and specific gaps in the literature that the new model, called the Intervention Innovations Team integrated conceptual model (IIT-ICM), seeks to address, followed by a brief description of the three theories/approaches that comprise the IIT-ICM and a rationale for their inclusion in the model, and a definition and description of the larger study, an optimization trial to test six intervention components grounded in the IIT-ICM, from which data for the present study are drawn. In Fig. 1 we present a schematic describing the steps leading to the present study.

### Barriers to engagement and gaps in the field that suggest the need for a new model

There is growing awareness that structural-level and systemic barriers drive racial/ethnic inequities in engagement along the HIV care continuum, but structural competency and structural salience are insufficient in many behavioral interventions [18–20]. Structural competence is defined as the trained ability to discern how issues defined clinically as symptoms, attitudes, or diseases, such as medication “non-compliance,” trauma, depression, or smoking, represent the downstream implications of a number of upstream decisions that shape powerful factors such as health care delivery systems, zoning laws, and urban and rural infrastructures [20]. Structural salience in behavioral interventions refers to the extent to which relevant upstream factors are reflected in behavioral intervention content and experienced by participants as reflecting their structural and systemic contexts, in contrast to conceptualizing behavioral challenges as a problem of individual decisions, faults and failings, or actions.

Further, there is a growing consensus that while public health research commonly focuses on race (e.g., differences between racial groups), it does not adequately attend to issues of racism, including systemic racism [18]. Systemic or institutionalized racism is a form of racism embedded through laws and regulations within society or organizations [21]. Examples of systemic racism for AABL-PLWH include factors that also affect AABL populations as a whole and include disproportional targeting of AABL persons by criminal justice entities, poor-quality health care available in the neighborhoods where AABL populations are concentrated, financial entitlement and benefit levels that keep people in chronic poverty, and unstable and/or low-quality housing and homelessness [22]. Other examples include pervasive surveillance mechanisms, such as probation, parole, and supervised supportive housing, which undermine dignity, autonomy, and self-determination among AABL persons [14]. Chronic poverty is another systemic factor that creates competing priorities related to survival needs such as food insecurity and also makes AABL-PLWH vulnerable to “diversion” of HIV medication; that is, the selling of HIV medication to pharmacies who solicit and compensate PLWH for prescriptions, although it is illegal for pharmacies to do so [23]. Behavioral interventions generally cannot change systemic or structural factors, but can acknowledge, understand, and address them and seek to circumvent or eliminate structural barriers to health outcomes.

AABL-PLWH generally express satisfaction with their individual health care providers, but evidence dissatisfaction with the health care and social service systems and care settings [24]. Substance use at both non-hazardous and hazardous levels, past and present, are very common among AABL-PLWH [25] but treatment for hazardous substance use among AABL-PLWH has historically been influenced by the abstinence-only model, and by punitive approaches that are not generally acceptable to or effective for AABL-PLWH [26]. AABL-PLWH report that approaches guided by harm reduction and those that enhance dignity, communicate non-judgment, and support personal autonomy are generally lacking in health care settings, but sorely needed [14, 27]. Support for autonomy is also a core element of self-determination theory, as we describe in more detail below.

In addition to substance use, PLWH’s HIV-related health behaviors exist within a constellation of what can be construed as potential harms to themselves and society, such as declining or taking long breaks from HIV medication, or idiosyncratic HIV medication dosing schedules (e.g., not taking medication on weekends). HIV and substance use treatment are similar in that treatment expectations are commonly absolute, leaving little room for individual autonomy. For example, the Centers for Disease Control and Prevention (CDC) recommends that PLWH initiate HIV medication immediately after diagnosis and then take “every dose, every day” to sustain HIV viral suppression [4]. Similarly, complete abstinence from substance use has historically been the predominant goal in most treatment settings [26]. There is clearly substantial variability in PLWH’s approaches to HIV management, yet clinical settings may not provide opportunities to discuss and explore those personal decisions to maximize health and wellbeing [9, 13–15].

Messages consistent with harm reduction appear relatively uncommon in HIV treatment settings [28]. However, perfect adherence to HIV medication is *not* required to achieve HIV viral suppression [4]. Current HIV medication regimens are highly effective and PLWH can achieve viral suppression with 80–90% adherence, or even lower depending on the regimen [4]. But this more pragmatic approach is not yet incorporated into the CDC’s definition of adherence (“every dose, every day”), and the CDC exerts a powerful influence on HIV care system policies and provider behavior. Thus, there may be utility to extending the harm reduction approach to include individuals’ health care and behavioral decisions about whether or how often to take HIV medication, and how to manage other aspects of their lives and relationships in ways that might reduce or eliminate harms to the self or others, including regarding substance use. Overall, AABL-PLWH do not experience most health care encounters as supporting their individual autonomy, but such support may be useful for treatment engagement [9, 13–15].

AABL-PLWH experience stigma related to HIV [29], certainly, as well as related to sociodemographic characteristics such as race/ethnicity, social class, and sexual/gender minority status, and this stigma emanates from a wide range of sources including social networks and institutional settings [30, 31]. Stigma experienced (or feared) in health care and social service settings related to AABL-PLWH’s substance use patterns impedes access to appropriate substance use treatment and medical care [30, 32]. Stigma further contributes to AABL-PLWH experiencing a general lack of social support from family and peers, and to extreme social isolation and self-isolation, all of which impede quality of life and engagement along the HIV care continuum [33]. Stigma can be considered intersectionally; that is, as interconnected social categories linked to overlapping and interdependent systems of discrimination or disadvantage [34]. Recently the field has moved to conceptualizing intersectionality as interlocking systems of oppression rather than intersections of personal identity [34], but integrating this perspective into intervention science is a new area of inquiry.

Other well-known barriers to engagement along the HIV care continuum for AABL-PLWH include fear and distrust of HIV medications, and of the health care system and medical settings [35]. Counter-narratives about the causes and treatments of HIV (called conspiracy theories in some cases) are another aspect of distrust [15, 35]. Distrust and counter-narratives are not generally explicitly addressed in interventions or health care encounters because clinicians may believe it is counter-productive to discuss these types of beliefs. Yet, these beliefs are common among AABL-PLWH and there may be utility in eliciting and exploring them to foster engagement, clarify health decisions, and build mutual trust [9, 15, 36]. Other barriers to the continuum include substance use and mental health concerns, which may produce competing priorities [13, 37–39]. Motivation to take HIV medication varies over time, but even when AABL-PLWH are ready to initiate HIV medication with high levels of adherence, motivation alone is commonly insufficient to overcome these multi-level barriers described above [40], suggesting the need for multi-level perspectives. These gaps further support the need for a new conceptual model, since no model to date has incorporated an emphasis on structural/systemic factors salient to AABL-PLWH, along with other core elements with the potential to engage AABL-PLWH and provide them with intervention and service approaches tailored their structural and cultural contexts and psychosocial needs in a manner they find highly acceptable.

### Brief description of the three theories/approaches that comprise the IIT-ICM

IIT-ICM was developed by our inter-disciplinary research team in an iterative process. To create the IIT-ICM, we reviewed the relevant literature including our own past research with AABL-PLWH [9, 13–15], identified gaps in existing treatment models and research studies (as reviewed above), decided whether an entirely new theory was needed or whether integrating existing complementary theories would have utility (and settled on the latter approach), selected the theories/approaches to be integrated based on the constellation of factors that impede engagement along the HIV care continuum reviewed here and gaps in the field, and articulated the core elements and key characteristics of the new model. When taken together, we maintain the three theories/approaches selected, namely, critical race theory, harm reduction, and self-determination theory, have areas of congruence and complementarity, and each theory or approach has aspects that may strengthen the others, potentially combining synergistically to create a new and useful tool. The three theories/approaches have particular salience for addressing racial/ethnic inequities, and have not been integrated previously.

Critical race theory is designed to illuminate contemporary racial phenomena, expand the public health discourse about the individual and social effects of institutionalized racism, and challenge racial hierarchies, including White cultural supremacy [41]. Critical race theory maintains that while systemic racism is less visible than individual racism, it is just as, if not more, influential [41]. Further, it points out that racism is baked into the fabric of society and therefore difficult to study [41]. Critical race theory argues for the importance of "centering the margins" and focusing on the non-dominant group’s lived experiences within the context of systemic racism (not just focusing on race). Moreover, critical race theory highlights the importance of counter-narratives and uncovering resistance and resilience found in AABL communities. Critical race theory was selected as a core element of the IIT-ICM to guide the identification and understanding of structural and systemic barriers to health, and underscore the roles of systemic racism, counter-narratives, and resistance and resilience in health behavior for AABL-PLWH. (We wish to note that the present study emphasizes aspects of critical race theory relevant to behavioral research but does not provide a comprehensive overview of the theory.)

Harm reduction is a conceptual framework and set of practices that focus on the minimization of the physical, social, and legal harms that may affect people who use drugs and to society as a whole as a result of drug use [42]. At its core, harm reduction supports the dignity and autonomy of people who use drugs without judgment. Harm reduction is also a movement for social justice built on a belief in, and respect for, the rights of people who use drugs [43]. A harm reduction perspective allows clinicians and drug users to work together to establish goals and objectives to reduce drug-related harm, based on the notion of an individual’s right to self-determination [44]. Harm reduction approaches have been found acceptable and effective in numerous past studies [45]. Because harm reduction is a way of viewing the impact of drug use on a person’s psychosocial functioning, that is, harm reduction is a perspective, it is not bound to any one behavioral change technique or therapeutic theory of action [46]. Harm reduction was originally developed as an approach to reduce drug-related harms. More recently the harm reduction perspective has been applied to other types of public health concerns, such as mental health distress [47]. Harm reduction was selected for inclusion in the IIT-ICM to underscore the importance of a non-judgmental and non-coercive approach to any positive behavior change related to HIV, substance use, or any other health behavior that AABL-PLWH wish to examine or change.

Self-determination theory is a macro theory of human motivation and personality that concerns the innate and fundamental needs for autonomy (people need to feel in control of their own behaviors and goals), competence (people need to gain mastery of tasks and learn different skills), and connection or relatedness (people need to experience a sense of belonging and attachment to other people) [48]. The most volitional and highest quality forms of motivation emerge when these three needs are supported by the larger environment, and self-determination theory proposes that the degree to which any of these three psychological needs is unsupported or thwarted within a social context will have a serious detrimental impact on wellness in that setting [49]. Indeed, autonomous/intrinsic forms of motivation are generally more effective in predicting health behavior than non-self-determined, external, or controlled forms [50]. Self-determination theory was selected as a core element of the IIT-ICM in light of the need for intervention approaches that support autonomy among AABL-PLWH, in order to foster durable, autonomous/intrinsic motivation for change and improve the quality of clinical encounters [14, 27], along with an emphasis on social relationships and the need to build competencies in HIV management. In practice, approaches to support autonomy are comprised of behaviors on the part of the “person in authority” such as a health care provider or behavioral interventionist that include providing explanatory rationales and pertinent information, acknowledging another’s feelings including negative feelings, relying on non-controlling language, minimizing pressure and control, emphasizing choice, and providing opportunities for choice [51–53].

Self-determination theory is an accepted theoretical underpinning of motivational interviewing [54]. Motivational interviewing is an evidence-based directive and collaborative counseling approach for behavior change that elicits participants’ values, perspectives, and questions, identifies ambivalence and discrepancies, and corrects misinformation with permission, to thereby foster durable intrinsic motivation and readiness for change [54, 55]. In reviews and metanalyses, motivational interviewing interventions have been found effective at clinically significant levels for a range of health behaviors [56–58]. Motivational interviewing has been found to be particularly effective with AABL populations compared to White populations [56]. As a non-coercive, strengths-based, and autonomy-supportive approach, it may have utility in particular when health beliefs and emotions such as distrust/fear impede behavior change [9, 15, 36].

### Description of the IIT-ICM

**Fig. 2:**
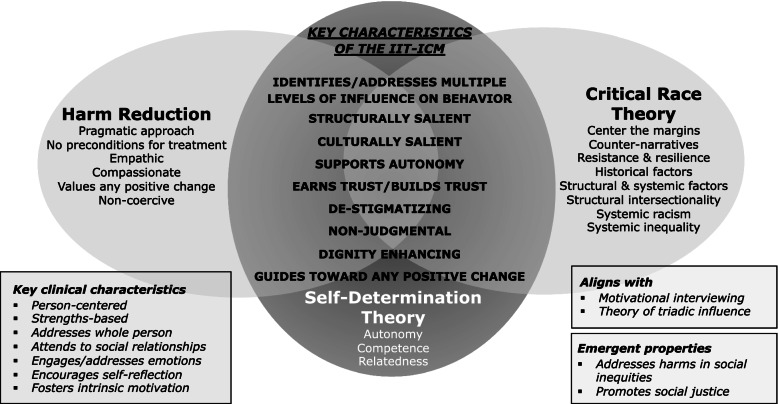
IIT-ICM core elements for ICM1

In Fig. 2 we present the results of this conceptual model-building process; namely, the core elements (the three theories/approaches) and key characteristics of the IIT-ICM. The IIT-ICM also yields key clinical characteristics; that is, important aspects of the intervention content and clinical encounter, some of which are under-emphasized in existing behavioral interventions (e.g., address emotions, encourage self-reflection, foster intrinsic motivation). As shown on Fig. 2, the IIT-ICM aligns with the motivational interviewing counseling approach. The IIT-ICM calls for an analysis of influences on behavior at multiple levels. Thus, as a means of organizing multi-level barriers to a health behavior, the IIT-ICM aligns with the theory of triadic influence [59]. The theory of triadic influence is a comprehensive multi-level social-cognitive theory that highlights the relevance of simultaneous structural-, social-, and individual/attitudinal-level streams of influences on behavior [59]. Emergent properties of the IIT-ICM include an intention to ultimately address harms in social inequities and promote social justice*.* In fact*,* one leader in HIV research recently noted that ending the HIV epidemic depends as much on social justice as on HIV medications [60]. As a new model, we anticipate that the IIT-ICM will be modified and refined as it is applied in research.

### Description of the optimization trial (field name: the Heart to Heart 2 project)

The present study uses data from a larger research study, an optimization trial grounded in the multiphase optimization strategy (MOST) framework [61]. MOST is an engineering-inspired framework for intervention development [61, 62]. To describe the MOST framework, we first contrast a typical study grounded in MOST with the classical approach. In the classical approach to intervention development, we typically create a behavioral intervention comprised of multiple components (e.g., health education, counseling, and reminder calls), called a packaged intervention. This packaged intervention is tested in a randomized controlled trial. The randomized controlled trial will provide information on intervention effectiveness (or not), but even if the intervention is found effective, the randomized controlled trial design cannot provide information on which of the intervention components contributed to effects or if all the components were active. If the intervention is not found effective, this design cannot provide information on whether some components were useful, or if the effect of one component cancelled out another. In contrast, in the MOST framework, highly efficient designs such as factorial experiments are used to estimate the individual and combined effectiveness of a set of separate intervention *components*. Then, a multi-component intervention can be created or “optimized” from these promising components. An optimized intervention is one that meets pre-specified criteria, such as the most effective combination of intervention components that can be carried out in x hours or for x dollars, or the most cost-effective combination of components. The effectiveness of this optimized intervention can then be confirmed in a randomized controlled trial.

The optimization trial carried out by our research team had the field name the “Heart to Heart 2” project, and was implemented in New York City between 2017–2021 [61, 62]. To prepare for the trial, six behavioral intervention components were developed, grounded in the IIT-ICM. (The specific steps we recommend for designing a behavioral intervention guided by the IIT-ICM are presented below in the Implications section of the Discussion). The behavioral intervention components are described below in the Methods section. The optimization trial focused on AABL-PLWH who were poorly engaged along the HIV care continuum, specifically, those who did not engage in HIV care at recommended levels and who had non-suppressed HIV viral load. It used an efficient fractional factorial experimental design comprised of 16 experimental conditions to test the efficacy of five separate intervention components. Further, a core intervention was received by all participants (thus six components total were administered). Participants were randomly assigned to one of the 16 experimental conditions. Each condition was comprised of a unique combination of intervention components (typically participants were assigned to receive 3–5 components including the core intervention). HIV viral suppression assessed by laboratory report was the optimization trial’s primary outcome. To date, participants have engaged in the optimization trial’s intervention components, and the intervention optimization process is ongoing. The trial is described in more detail elsewhere [61].

The present qualitative study explores participants’ experiences in and perspectives on the acceptability, feasibility, and effects of the optimization trial, including uncovering and describing the role of the new IIT-ICM in fostering acceptability, feasibility, and effects.

## Methods

### Overview

The present study was qualitative and exploratory and took an ethnographic/phenomenological qualitative approach [63]. It used semi-structured in-depth interview data collected as part of the optimization trial. We also present descriptive quantitative data on participants’ sociodemographic and background characteristics and on study acceptability. The optimization trial was registered with ClinicalTrials.gov (NCT02801747). Participants gave signed informed consent for study activities. The study was approved by the Institutional Review Board at the New York University Grossman School of Medicine.

### Eligibility criteria for the optimization trial

The optimization trial’s inclusion criteria were 1) age 18 – 65 years; 2) African American or Black race and/or Latino/a or Hispanic ethnicity; 3) HIV diagnosed for at least 6 months; 4) HIV antiretroviral therapy adherence less than 50% in the past six weeks (assessed by self-report) *and* non-suppressed HIV viral load based on a laboratory report; 5) sub-optimal engagement in HIV care (operationalized as less than one visit in every four month period in the past year *or* > two missed visits without prior cancellation in the past year) assessed by self-report; 6) resides in the New York City metropolitan area; 7) able to conduct research activities in English or Spanish; 8) willing to provide a blood specimen at screening to assess HIV viral load; and 9) willing to be randomly assigned to 1–5 intervention components and receive the core intervention. Participants were found eligible for the optimization trial if they met all the inclusion criteria.

### Staff and training

Study staff were diverse with respect to age, gender, race/ethnicity, and HIV status and had bachelor’s or master’s degrees in the social sciences (e.g., social work, public health, and psychology). All had past research experience with the population of AABL-PLWH. To promote high rates of engagement in the study, all staff members were trained in the IIT-ICM and the study ethos that aligned with the IIT-ICM which emphasized the importance of structural/systemic factors, respect for autonomy and personal decisions regarding HIV medication use, and on providing an overall high-quality experience (e.g., staff members remembering participants’ names, flexibility in rescheduling, prompt compensation, acknowledgement that participants’ time is as valuable as staff time, and refreshments provided in the waiting area and during sessions or groups). Cultural competency in health care describes the ability of systems to provide care to clients with diverse values, beliefs, and behaviors, including the tailoring of health care delivery to meet patients' social, cultural, and linguistic needs. Cultural competency is more commonly attended to in intervention development and clearly an important aspect of behavioral interventions. Thus, staff were trained to be both structurally and culturally competent to foster trust, study engagement, and the behavior change process.

#### Brief description of the behavioral intervention components

The core intervention was a single, brief (< 60 min) individual session with two main goals. The first goal was to provide or reinforce basic health education on HIV management that comprised the standard of care (e.g., the expected frequency of HIV care visits and HIV medication adherence patterns). The second was to introduce the participant to the study ethos grounded in the IIT-ICM to thereby begin to foster a constructive relationship between the study and the participant to support engagement in other intervention components. Component A: motivational interviewing sessions (four sessions, 60 min, each) was designed to use specific motivational interviewing techniques to address salient, including culturally salient, health beliefs (e.g., outcome expectancies, self-efficacy, medical distrust) and emotions (e.g., concerns/fears of HIV medication) to foster durable intrinsic motivation for behavior change. Component B: pre-adherence skill building (four months’ duration) was designed to help participants build behavioral skills to manage HIV medication adherence such as habits while attending to cultural and structural factors that can impede adherence (e.g., lack of private living space). Component C: peer mentorship (four months’ duration) was facilitated by a “successful” peer mentor (i.e., a PLWH demographically similar to study participants who had consistently engaged in care and was taking HIV medication with high levels of adherence) and sought to provide peer modeling of and shape peer norms regarding HIV management (primary goals), and provide social support and combat stigma (secondary goals). Component D: focused support groups (six groups, 90 min. each) were designed to provide social support and reduced stigma regarding care and HIV medication use, including culturally salient factors that impede engagement such as medical distrust and fear and structurally salient factors (poor-quality housing, pharmacies buying medications from PLWH). Component E: navigation was designed to identify and ameliorate structural barriers to care and HIV medication. In this factorial design, all components had two “levels.” Components A-D’s levels were off/on (participant did not receive/participant did receive), and Component E’s two levels were short navigation (three months) vs. long navigation (six months). The intervention components were flexible and individualized (e.g., as a counselor-delivered intervention content was shaped and modified in the session to meet participants’ needs, and intervention manuals contained alternate exercises depending on participants’ desire to take HIV medication or not). The components did not assume that participants wished to or were ready to take HIV medication at the present time.

### Procedures for the optimization trial

#### Recruitment into the optimization trial

The recruitment approach for the optimization trial comprised a hybrid sampling strategy that included peer-to-peer recruitment; direct recruitment by study staff members in HIV service, HIV housing, and other community-based organizations; and advertisements in a local free newspaper. Peer recruitment was the primary sampling approach. Peer recruitment was tracked with a coupon system that linked the recruiter to the recruit, and recruiters received modest compensation for recruitment ($15/recruit). Most enrolled participants were recruited by peers (75%); 9% were recruited through newspaper ads, and 16% through other means.

#### Screening, enrollment, intervention activities, and follow-up assessments

Participants were screened for eligibility after providing informed consent. Screening included assessment of HIV viral load levels via laboratory report obtained from a commercial laboratory. Those found eligible for the optimization trial provided signed informed consent, and completed a structured baseline assessment battery lasting 60–90 min. The baseline was conducted in the Research Electronic Data Capture (REDCap) platform. REDCAP is a cloud-based platform for data capture designed for clinical research [64, 65]. After completing the baseline assessment, participants were randomly assigned to an intervention condition using a randomization table created by the study’s biostatistician and located in REDCap. Participants were randomly assigned to one of 16 different experimental conditions, each comprised of the core intervention and a different combination of the five behavioral intervention components [61]. The period during which participants engaged in intervention activities ranged from 4 to 8 months. Regarding the timing of intervention activities, the core intervention and Component E: Navigation were provided first, to begin to address structural barriers to engagement along the HIV care continuum (all participants received core intervention and either 3 or 6 months of navigation). Component A: Motivational interviewing sessions were scheduled next for those randomly assigned to receive that component. Component C: Peer mentorship and Component D: Focused support groups could be administered in the same time period (although not on the same days), and Component B: Pre-adherence skill building was provided last for those assigned to receive it.

Participants received follow-up assessments at 4-, 8-, and 12-months post-baseline, comprised of a structured interview and HIV viral load test at a commercial laboratory (at 8-, and 12-months). Thus, participants were enrolled in the optimization trial for 12 months. In-person study activities took place in confidential offices at a project field site in lower Manhattan in New York City. Participants were compensated $15 for a screening interview, $15 for providing the blood specimen for HIV viral load testing, $25 for baseline and follow-up assessments, and $25 for each intervention session or activity, along with funds for local round-trip public transportation.

#### Effects of the COVID-19 pandemic on the optimization trial

The first case of COVID-19 was diagnosed in New York City on March 1, 2020. On March 12, 2020, in-person activities with human subjects were suspended at New York University, although virtual activities could continue with IRB-approval. At this point in the study, 241/512 (47%) had completed participation in the study, with the remainder still scheduled to attend intervention activities and/or follow-up assessments. With the exception of focused support groups, which were not feasible given participants’ lack of smartphone and computer access [66], remaining intervention components and follow-up assessments were carried out in a virtual format. Because we could not escort participants to a commercial laboratory since travel to laboratories was restricted, we requested that participants provide a recent laboratory report from their existing HIV care clinic for which they would be compensated $30. If that was not possible, participants could present independently to a commercial laboratory and the research study would compensate the lab for the viral load test. Yet because early in the pandemic HIV services and travel were disrupted due to the public health order to remain at home, it was commonly challenging for participants to carry out laboratory tests or obtain reports. The COVID-19 pandemic resulted in some delays in engagement in study activities and modestly reduced the proportion that provided HIV viral load results.

### Procedures and materials for the present study

#### Selection of participants for qualitative interviews

A total of 2–4 participants from each of the 16 experimental conditions were randomly selected for two qualitative, semi-structured, in-depth interviews, one early in the study (within 5–7 months of enrollment) and another at study completion. The qualitative interviews sought to understand participants’ experiences living with HIV and with the optimization trial. The qualitative interviews were audio-recorded and professionally transcribed verbatim. Individuals were compensated $25 for each qualitative interview, along with funds for local round-trip public transportation. A total of 46 participants engaged in the first qualitative interview and 32 of these also completed the second interview (thus 70% participated in both interviews). Interviews were conducted in-person at the study field site prior to the COVID-19 restrictions, and on the phone after restrictions on in-person activities were implemented.

#### Qualitative semi-structured interview guide

The in-depth interviews were guided by a semi-structured interview guide developed by the research team, which included experts on AABL-PLWH and the HIV care continuum. The guide was based on a review of the literature and guided by the IIT-ICM. The guide was pilot tested prior to administration and refined. It was structured as a series of questions and prompts, starting with more general questions and moving to more specific ones. Throughout the interview process, the interview guide was updated to reflect newly emergent concepts (e.g., feeling pressured to take HIV medication and its effects). The guide was organized into sections: 1) general experiences with the study (e.g., To start off, what was it that led you to agree to participate in the study? What stands out to you most about the project so far? What have you like? Disliked?) 2) emotional or behavioral effects of study participation or recent changes concurrent with study participation, if any (e.g., Have you taken HIV medications since you joined the Heart to Heart 2 study? It’s OK if you haven’t. We just want to understand what’s going on with you now. Why or why not? What factors played a role in your deciding to take HIV medications at this time, whether related to the Heart to Heart 2 study or other factors? Since you’ve been involved with the study, has anything changed about the way you think about HIV medication?). We also explored participants’ perspectives on the individual intervention components they were assigned to receive, which will be presented in a future study. The relevant sections of the interview guide are provided as Supplemental Material.

#### Qualitative data analysis

The strategy used a directed content analysis approach that was both inductive and theory-driven [67]. First, a primary researcher trained in medical anthropology analyzed interview transcripts in the Dedoose platform and developed an initial start-code list and operational definitions for each code, informed by the theoretical and conceptual perspectives guiding the study [68]; namely, the underlying IIT-ICM including barriers to and facilitators of engagement along the HIV care continuum as structural-, social-, or individual/attitudinal-level influences. Thus, codes included those related to culture and race/ethnicity (e.g., experiences of discrimination, medical distrust, counter-narratives), substance use management, and autonomy, competence, and relatedness, as well as about other factors that promote or impede engagement along the HIV care continuum (e.g., housing, mental health distress, and poverty). Then, the primary analyst coded approximately 20 transcripts using the start-code list. Next, two additional trained qualitative researchers coded a subset of the interview transcripts and met frequently with the primary data analyst. Codes were further refined and elaborated upon, and discrepancies were resolved by consensus. After resolution of discrepancies, each transcript was then recoded using the final coding frame. Then, in an iterative process and in collaboration with an interpretive community made up of members of the research team, codes were combined into larger themes and sub-themes [69, 70].

Regarding positionality and methodological rigor, the research team was made up of cisgender men and women, and transgender and genderqueer people, from White, African American/Black, Asian, and Latino/a racial/ethnic backgrounds. The primary data analyst was a member of the research team trained as a medical anthropologist and experienced with HIV research, including with this subpopulation of AABL-PLWH. Positionality challenges related to sex, gender, race/ethnicity, power, health, socioeconomic status, and privilege were intentionally addressed throughout the data collection process through reflection and training, which focused on how these types of issues might impact the interviewing process and data analysis [71, 72]. Although we used the random sampling method for the qualitative interview based on the demands of the optimization trial, we attended to issues of maximum variation in sample characteristics [73] as one aspect of trustworthiness [74]. Methodological rigor of the analysis was further maintained through an audit trail of process and analytic memos and periodic debriefing with the larger research team, which included PLWH and experts in long-term HIV survivorship and HIV medication adherence, as well as member checking with AABL-PLWH; feedback from the member checking was incorporated back into the results [63].

#### Quantitative measures

We assessed age, sex assigned at birth, gender identity, sexual minority status (i.e., identifies as gay, lesbian, bisexual, queer, or other non-heterosexual identity), race/ethnicity, housing status, history of incarceration (yes/no), indications of extreme poverty (how often unable to pay for necessities in the past year and food insecurity) with structured instruments developed for populations in high-risk contexts [75]. The Adverse Childhood Experiences Scale- revised (ACES-R) 14-item scale was used to assess early life experiences such as peer victimization, neighborhood disorder, physical abuse, neglect, and sexual abuse [76]. We used a version of the HIV Cost and Services Utilization Study (HCSUS) [77] instrument to assess years since first HIV diagnosis; years since first initiated ART; number of months since last HIV medication dose (if not on HIV medication at screening). HIV viral load was assessed with a laboratory report and suppressed viral load was coded as < 200 copies/mL. Substance use patterns were assessed by the World Health Organization Alcohol, Smoking and Substance Involvement Screening Test (WHO ASSIST) [78]. Using established thresholds, symptoms of depression were assessed with the Patient Health Questionnaire depression module (PHQ- 9) and coded as likely depression (yes/no) [79]. The Generalized Anxiety Disorder scale (GAD-7) was used to assess symptoms of anxiety and coded as likely anxiety (yes/no) [80]. The Primary Care PTSD Screen was used to assess symptoms of PTSD, coded as likely PTSD (yes/no) [81]. Study acceptability was assessed using the 12-item Client Satisfaction Survey [82]. Feasibility was defined as the proportion of participants who completed planned study activities.

#### Quantitative data analyses

We used descriptive statistics to summarize socio-demographic and background characteristics and study acceptability using R [83].

## Results

Participants’ sociodemographic and background characteristics are found in Table 1. Participants were 49 years old, on average (SD = 9 years). Most (78%) were assigned male sex at birth. Approximately a third (33%) were sexual and/or gender minorities. The majority (76%) were African American or Black and the remainder were Latino. Rates of adverse childhood experiences ranged from 0–14 (mean = 4, SD = 3 experiences). Indications of low-socioeconomic status and extreme poverty were common: Only 17% were employed, nearly half (46%) had run out of funds for necessities at least monthly in the past year, and most (85%) experienced food insecurity often or sometimes in past year. Approximately half (52%) were not stably housed. Participants had been diagnosed with HIV 19 years ago, on average (SD = 7 years). All had taken HIV medication in the past. The longest duration of sustained HIV medication use was 45 months (SD = 63 months). Current substance use was common: approximately half (54%) used alcohol at a moderate-to-high-risk level based on The World Health Organization ASSIST measure scoring criteria, 61% used cannabis at a moderate-to-high-risk level, and 63% used cocaine or crack at a moderate-to-high-risk level. Less than 10% injected drugs in their lifetimes or the past three months. Most (78%) engaged in substance use treatment in the past. Approximately one third of the sample or less reported likely depression, anxiety, or post-traumatic stress disorder (PTSD). A total of 40% evidenced suppressed HIV viral load assessed via a lab report at the 8- and/or 12-month follow-up period.

**Table 1:** Participant sociodemographic and background characteristics (*N* = 46)

	**M (SD) or %**
Age (range 23 – 62 years)	48.9 (8.74)
*Sex assigned at birth*	
Female	21.7
Male	78.3
Sexual and/or gender minority status	32.6
Transgender gender identity, gender fluid, gender non-conforming	4.3
African American or Black (non-Latino/Hispanic)	76.1
Latino or Hispanic	21.7
Stable housing (has their own home or apartment, including funded by government programs or benefits)	47.8
Adverse Childhood Experiences (ACES-R) score (range 0–14)	3.56 (3.33)
*Indications of low socioeconomic status and extreme poverty*	
Working full-time or part-time off-the-books or on-the-books	17.4
Ran out of funds for necessities monthly or more in the past year	45.7
Food insecurity often or sometimes in past year	84.8
Engaged in transactional sex – past year	17.4
*HIV-related factors*	
Years since HIV diagnosis at enrollment (range 3.0—30.0 years)	18.6 (7.18)
Median [Q1, Q3]	18.5 [13.3, 24.0]
Took HIV medication in the past	100
Times stopped/started HIV medication in the past (range 0—100 times)	10.6 (17.8)
Longest duration of sustained HIV medication, in months (range 0–264 months)	45.0 (62.7)
*Psychosocial risk and protective factors*	
Alcohol use at a moderate-to-high-risk level	54.3
Cannabis use at a moderate-to-high-risk level	60.9
Cocaine or crack use at a moderate-to-high-risk level	63.0
Use of other drugs (not including alcohol, cannabis, cocaine/crack) at a moderate-to-high-risk level	28.3
Never injected drugs	87.0
Injection drug use lifetime, but not in the past 3 months	6.5
Injection drug use – past 3 months	6.5
Participated in substance use treatment in the past	78.3
Likely depression	21.7
Likely anxiety	10.9
Likely PTSD	34.8
*HIV viral load*	
HIV viral load level at enrollment (log_10_ transformed)	4.28 (0.970)
Suppressed HIV viral load at 8- and/or 12- month follow-up assessment	40.0

In Table 2 we present participants’ acceptability ratings of aspects of the study overall at the final follow-up assessment for the entire sample. Acceptability ratings were high (> 70%). Regarding feasibility, despite disruptions due to the COVID-19 pandemic as described above, assessment follow-up rates were high: 83.4% completed the 4-month, 81.1% completed the 8-month, and 80.7% completed the 12-month follow-up assessment.

**Table 2:** Acceptability ratings at the final follow-up assessment (*N* = 411)

	%
Overall, I think the activities and services in the Heart to Heart 2 project are good to excellent	90.0
The information I have received in the project has been helpful or very helpful	92.2
The staff of the project have answered my questions most of the time to all the time	91.0
The project staff treats me like I am an individual with unique needs and concerns most times to all the time	90.5
The project staff respects my privacy most times to all the time	90.8
The project staff understand the needs of people of my racial, ethnic, or cultural group most times to all the time	87.1
(If sexual/gender minority status) The project staff understand the needs of people who identify as LGBTQ (lesbian, gay, bisexual, transgender and queer)	82.0
(If female) The project staff understand the needs of women most times to all the time	92.1
(If < 36 years old) The project staff understand the needs of younger people (< 36 years old) most times to all the time	78.5
(If > 50 years old) The project staff understand the needs of older people (≥ 50 years old) most times to all the time	88.5
Participation in the Heart to Heart 2 project affected my decision to regularly attend HIV medical care somewhat to a great deal	76.2
Participation in the Heart to Heart 2 project affected my decision about whether or not to start HIV medication somewhat to a great deal	71.8

### Overview of results

Participants described managing a confluence of recurring challenges and crises, all exacerbated by chronic poverty, throughout the time they were enrolled in the optimization trial. These commonly included homelessness and/or poor quality or unstable housing, relationship instability, involvement with probation and parole systems, histories of trauma exacerbated by current trauma, underlying physical health comorbidities in addition to HIV, challenges with substance use management and, in some cases, hazardous substance use, the need to sell HIV medication to meet basic needs, and severe mental health distress. The high prevalence of structural/systemic barriers such as poverty and housing challenges is consistent with the IIT-ICM, which emphasizes attention to structural factors. Nonetheless, even in this challenging structural/systemic context, the majority of participants were still able to reflect on their own strengths and resilience. Further, we found participants maintained both the desire and ability to make changes in health behavior and other aspects of their lives consistent with their own values, including within the “safe space” that they reported the optimization trial provided. Participants overwhelmingly emphasized that the trial provided a space within which they felt welcomed, individually cared for, and, ultimately, within which they were able to reflect on both their emotional and physical health, including their management of substance use, anxiety and depression, personal relationships, HIV, underlying health conditions, and in many cases their willingness, ability, and/or desire to re-initiate HIV medication or to increase HIV medication adherence in order to achieve HIV viral suppression. Even when participants did not elect to initiate HIV medication or increase the number of HIV medication doses they were taking during the trial, they reported that engagement in project activities typically resulted in other types of psychosocial, emotional, or tangible improvements in their lives. Consistent with the IIT-ICM, participants were encouraged to stay engaged in the study even if they elected not to focus on or work toward the study’s primary outcome, HIV viral suppression, without experiencing pressure or judgment from project staff. Generally, participants engaged in frank discussions of their health decisions (e.g., not taking HIV medication) and contextual challenges (e.g., selling HIV medication, substance use) with project staff, including discussing those behaviors not typically considered socially desirable or socially acceptable, which may reflect the IIT-ICM and its emphasis on non-judgment, harm reduction, and personal autonomy.

We organized results into the following four inter-related themes: the importance of feeling understood and validated as a whole person and in their structural/systemic context; experiences of trustworthiness and trust; opportunities for self-reflection on a range of topics and its effects; and support of personal autonomy and its effects on motivation and decisions. The importance of nonjudgment was prominent in each theme. We also provide findings on the context of participants’ lives in the sections that follow and highlighted the ways the results appear to reflect the IIT-ICM. In reporting the qualitative results, we present findings pertaining to participants’ experiences in the optimization trial as a single entity (regardless of what intervention components they were assigned to receive), with some references to specific intervention components for clarity. This is because participants did not generally report experiencing the study as being made up of individual components; instead, they experienced their time in the optimization trial, which they refer to as the Heart to Heart 2 project, as a whole. Gender-neutral pronouns (they/them/theirs) were used in the sections that follow because we did not assess which pronoun series participants used to describe themselves. We used pseudonyms and changed or obscured identifying details to maintain participants’ confidentiality.

### The importance of feeling understood and validated as a whole person and in their structural/systemic context

Participants emphasized the important role that the contexts of their lives, as noted above, and the characteristics of institutional settings they commonly engaged in, had on behavior and wellbeing. First, they emphasized that social isolation was both chronic and extreme. They reported being generally unable to connect with healthcare, mental health, and other service providers in a manner that was non-transactional, non-judgmental, and meaningful, with one exception; namely, support groups at local community-based organizations oriented toward working specifically with PLWH. Overall, participants felt unwelcome and devalued in health care settings, and were typically hesitant to openly discuss issues such as mental health and substance use with healthcare providers and social service agencies. Participants reported that in many service settings, and particularly in HIV care settings, they frequently experienced feelings of invalidation and lack of individualized care, describing instances wherein they felt like a number, and were treated as “less than human.” This typically resulted in an erosion of their intrinsic motivation to prioritize themselves or their health, which in many cases led to feeling *less* motivated, rather than more motivated, to improve their HIV medication adherence patterns after engaging in health care settings.

In contrast, when asked to reflect upon their experiences during the optimization trial, participants typically discussed feeling understood as an individual, and, in many cases, as a whole person. They commonly reported that the trial was one of the first times they felt viewed in a professional setting as a complex person with well thought-out and worthwhile perspectives and needs. As shown in Fig. 2, the IIT-ICM emphasizes, in part, individualized care, participant dignity, and reduction of stigma. Participants indeed did commonly experience relationships with project staff as de-stigmatizing, mutually respectful, possibly dignity enhancing, and, in many cases, caring. This, in turn, fostered a clinical context in which they could explore aspects of their lives they generally experienced as underappreciated, disregarded, and stigmatized in most professional settings, in relative safety.

Upton, a Black, gay, cisgender male in their late 40 s, who had been living with HIV for 10 years, described the importance of their larger context and the cascading effect that poor quality housing could have on HIV medication adherence, by triggering depression.

I moved to this room, it’s horrible, in the Bronx. It’s very hot, you can’t cook, you have to buy every meal. […] It’s so much more expensive having to buy every meal, every day of the week. […] This place it kind of makes it hard, but I’m trying to keep on the up. Because I know if I go down [get depressed], then my adherence is going to get thrown off. […] If I get to my depression or I just – because there’s been times the [medication] bottle has just been sitting there, and I didn’t sell it, and I still didn’t take it. That’s just the only battle. And that’d be my mental health issues, and hopefully I don’t – I could stay on top of that. The weed helps. You know what I mean? I’m just being real.

Thus, Upton highlighted the importance of behavioral interventionists and care professionals eliciting and understanding the context of participants’ lives, including how poverty can create contexts such as poor housing quality that are not conducive to HIV medication adherence, and how entities in the larger environment, such as corrupt pharmacies that buy HIV medication from patients, interfere with health behavior. Further, Upton described a personal harm reduction approach to preventing their selling HIV medication to corrupt pharmacies: In order to prevent themselves from diverting their HIV medication, they would “like punch the foil open once I pick it up from the pharmacy.” This is because once the foil on the pill bottle was punctured, it would not be possible to sell the bottle, and Upton would be more likely to adhere to their medication regimen. They continued, “Even though I may say I’m not going to sell it, but – and even though there’s no thought of selling it, [I] punch it anyway.” Indeed, guided by the IIT-ICM, the intervention components in the optimization trial were designed to elicit and address or circumvent these types of structural and contextual factors, and prompt participants to develop harm reduction strategies.

Simone was a Black, heterosexual, cisgender female in their early 40 s who had been living.

with HIV for approximately 15 years, and who struggled with depression. Simone described a history of very difficult experiences with medical providers and managing health care systems, including numerous cases where providers pressed them to make certain medical decisions, but did not truly understand Simone’s needs. They described:

The doctor actually gave me a prescription that I'm allergic to [related to an egg allergy]. That means that you're not paying attention to my chart. Like these are the things that you go through [in HIV care settings], and if you're not an assertive person like I am, [if] you're not paying attention, [if] you're not reading these papers, [if] you're not reading what they're ordering, [if] you're not reading what you're signing, you're going to end up getting a flu shot that you're allergic to. So that just made me feel like, okay, you guys are just not [paying attention].

Simone had not taken HIV medication for over a year at the time they enrolled in the.

optimization trial. They described their experience in the trial as “personalized,” in contrast to the typical medical setting, and these personalized experiences contributed to their deciding to access treatment for depression and, sometime later, re-initiate HIV medication.

Hank, a Black, gay, cisgender male in their early 50 s, who had been living with HIV for approximately 10 years, contrasted their experiences in the optimization trial with typical health care setting encounters. They noted a marked lack of social and structural stigma in their interactions with project staff, consistent with the IIT-ICM:

And you know, just in passing I spoke to like the receptionist [at the project], the people that log them [participants] in, and you know, it's like you don't feel like a pariah, you don't feel like nobody's scared of you because you're HIV positive. It's like people talk to you like a real person, and that matters more than anything. […] People were treating me like I'm a person instead of a number. [...] Other than that, it's just, I just feel comfortable [at Heart to Heart 2]. Usually when you go to places like this [that serve PLWH], people make you feel like, you know, hands off. They don't make you feel comfortable at all. It's like totally psychological, and sometimes, you know, you ain't in the mood for that shit.

Relatedness (i.e., social relationships) is one important aspect of the IIT-ICM. Like with Hank, many participants described the ability to develop meaningful, ongoing connections with project staff as a critical part of their experiences with the optimization trial and the foundation, in turn, for their being willing and able to address a broad range of HIV medication-related struggles in a holistic manner.

On the other hand, participants commonly expressed frustration that their primary care providers and other social service providers were not better able to see their HIV care and HIV medication adherence struggles in a larger context. In particular, they experienced their providers as “dismissive” of a number of issues participants considered vital, such as housing insecurity, personal relationships, substance use, and legal problems. Participants reported providers appeared to see these types of concerns as peripheral or even unrelated to HIV management. Yet, participants were clear these issues were directly related to HIV care and medication, and the lack of such recognition commonly left participants feeling overlooked, frustrated, or rejected. In contrast, the IIT-ICM highlights the importance of understanding a participant or client’s larger context, including structural and systemic factors that may affect health behavior. Jared, a Black, heterosexual, cisgender male in their early 60 s, who was diagnosed with HIV at the age of 40 and who was struggling with medication adherence and substance use issues while in the trial, drew a stark contrast between the connections forged during their time with the optimization trial and with other settings. They repeatedly stressed the importance of feeling seen and heard by all project staff members alike:

Because the basic thing here with you all is that you listen. All of you all. Even you. I'm looking at you. You're listening to me. You ain't trying to blow me by. Anybody's trying to blow me by [I] would get up and say have a nice day. I'm like that. Quick. But you listen. I seen that when I first walked in. It's important. Because people are hurting, you never know. See, when you don't listen to someone, that – I have seen people in my life, I'll be honest with you, I've seen like six or seven people commit suicide because nobody listened to them [...] And that's why I love this place, because you'll listen. Every last one of you that work here, you're listening, and then you'll give back your feedback. I never got no bad [unhelpful] feedback from none of y'all.

Thus, Jared highlighted the importance of being “listened to” as a precursor to receiving feedback that might assist them in achieving their health goals. This experience of being listened to and heard may reflect the IIT-ICM in that participants’ larger contexts are considered a vital part of the clinical encounter. We found that when participants felt they were being genuinely listened to, the level of investment they felt by staff translated to the participant developing a deeper level of investment in themselves.

It follows that since the IIT-ICM highlights the importance of eliciting and understanding a participant as a whole person and in context, participants are more than just an “HIV positive person.” Results indicated that having a space where participants experienced individualized services that took the larger context into consideration, while also supporting autonomy, commonly fostered their abilities to see HIV and HIV medication as *aspects* of their lives, and therefore, to reduce the sense of being defined solely by their HIV status. Steven was a Black, heterosexual, cisgender male in their early 50 s, who had been living with HIV for 20 years, and for whom “going to a doctor is a reminder” of their HIV status. For Steven, the ability during the optimization trial to have an “open dialogue” with chances to “branch off” into other areas of their life presented an “unexpected opportunity” to connect the painful emotions related to living with HIV and their experiences in typical HIV service settings more directly with their ongoing HIV medication adherence challenges. In the following quote, they refer to the optimization trial in general, and to Component D: Focused support groups:

You come to a place where [you can engage with] people with the [HIV] virus and shit. And you're offered a chance with people with the virus to talk and feel relaxed and comfortable. There's not that many places that you can feel human, because you've got to understand: There are times you feel less than human, which is why a lot of people don't take their medication and everything, because they want to forget. They want to be – I'm just normal. I'm like everybody else. So, you don't take medication, and you don't present to doctors and do what you want to do to live that fantasy of being everybody else. And you put yourself in jeopardy. Truthfully, you're putting more people in jeopardy.

As with most participants, feelings of validation that their life was seen in context, that they were valued as a whole person, and of connectedness with the project staff allowed for more open and honest conversations about a range of issues directly and indirectly associated with HIV care and HIV medication adherence. Further, the sense of acceptance and connectedness that resulted from the intervention activities grounded in the IIT-ICM were reported to increase participants’ views on the acceptability of intervention components, as well as the likelihood that participants would continue to actively engage with the trial over time; that is, study feasibility.

### Experiences of trustworthiness and trust

As noted above, distrust of health care systems, HIV medications, and counter-narratives about the causes of HIV and its treatments are very common among AALB-PLWH, while distrust in actual health care providers is less prevalent. Yet, participants’ trust in a project and the project staff are critical for perceived acceptability of services, ongoing engagement and thus study feasibility, and effective clinical service provision. Trust is a central precursor to effective counseling interventions and productive patient-provider relationships, as noted above. The IIT-ICM was designed in large measure to create a research project that was worthy of trust and to build trust. In part this was addressed by eliciting and understanding the valid reasons for medical distrust and counter-narratives among AABL-PLWH, since medical distrust and distrust of research projects are related. Guided by the IIT-ICM, the project staff sought to build participants’ trust and communicate their trust in the participants to make their own health decisions, even in the context of medical distrust (which the project sought to elicit but not necessarily to question or change). Thus, to some extent trust between the project and participants was built by eliciting, discussing, and understanding the valid reasons for distrust and counter-narratives, and by the project expressing trust that participants would make the right decisions for themselves.

Participants frequently discussed the importance of trust and honesty in medical care and social services. They provided numerous examples of times when they either had or had not trusted or been honest with others, and also times when they had and had not been honest with themselves. Consistent with the intentions of the IIT-ICM, participants commonly experienced the optimization trial as an environment where they could speak openly and honestly about matters related to their physical and mental health care that, in the past, they typically felt were off-limits or that they avoided out of an abundance of caution. Of vital importance, participants noted they experienced being trusted and being seen as trustworthy in the trial. For example, they experienced project staff as assuming that they (the participants) were experts on their own health and could and should make their own health-related decisions. This perspective, in turn, contributed to feelings of confidence and self-efficacy with respect to these health decisions. In contrast, participants reported commonly experiencing great pain and frustration in health care settings in cases where they were not trusted to be expert on their own health. This theme of the importance of trust and trustworthiness was noted in discussions about relationships with HIV medical providers, project staff, and other support staff in participants' lives. Upton, introduced above, described a long history of significant mental health concerns and complicated substance use patterns, coupled with pervasive homelessness, which began when they disconnected from their family decades prior. Upton explained that all of these experiences made it very difficult to remain in medical or mental health care on a continuous basis, particularly over the past five or six years. They discussed how they had been selling their HIV medication when they needed money for necessities, and sometimes to purchase drugs. Upton described being able to discuss these types of experiences honestly and without judgment during the optimization trial, suggesting Upton found the project trustworthy. Upton described that those honest discussions in and of themselves helped to develop motivation for their personal HIV-related health goals:

Even when I didn’t take it [HIV medication] – I mean I remember them times – when was it? Especially late last year, early this year, January, when I actually didn’t take a bottle, and I was just totally honest – I think I had a meeting with [my navigator] right around the next day I think I sold the bottle.... And I didn’t feel judged. She said, “I’m so glad you’re honest and open with me about it.” I didn’t feel pressure or discrimination at all.

Similarly, Ronald, introduced above, described the optimization trial as a place where they learned a great deal about themselves, their decisions, and their motivations. They noted the environment provided space for them to be honest with the project staff, which led Ronald to become more honest with themselves, and also more honest with their health care providers and others in their life. They described feeling trusted by staff to be a reliable reporter of their own experiences, including related to substance use problems, and that experiencing that trust helped them to prioritize their own health:

[Heart to Heart 2 has] taught me a lot, man. It’s taught me that I need to be honest with myself. I need to be true with me about everything I do in life. Even with this young lady I met [a new romantic interest], even with myself. I should be number one in this world because without me, I can’t make anyone else happy. […] That’s just the outcome of life, man. I want to be somebody in this world. I want to leave a legacy. […] I want my family to say wow, at least he went bad and then he became someone and he did something [positive]. He left this for his nieces. That’s my plan.

Wallace, a Black, gay, cisgender male in their mid-40 s, who had been living with HIV for decades, described their life history as one of being in service to others, coupled with a lengthy history of substance use, unstable housing, and mental health issues. They saw the environment in the optimization trial as one that engendered a feeling of trust, which in turn enabled them to open up about their myriad life experiences in a way Wallace had never experienced before, reinforcing a sense of trust they were developing within:

[Being able to talk about things not directly related to my HIV medication] is what always happens when I come here. And that’s why I say it’s very helpful, because I don’t go anywhere else and talk like this. […] So, whenever I come here, I’m able to process some stuff that I don’t process anywhere else. I think that Heart to Heart creates a level of comfortability for people to be able to open up. Just the vibe and the spirit from your workers. […] And some places you go to it’s just very business and there’s a very fine line. […] Like here, I come, and I just feel like I’m able to talk to you guys. I can't explain it. Maybe it’s just the vibe here is much more laid back than some of other places. So, you don’t feel as though you’re restricted well, I’m here for this and that’s that. […] And some places it’s like, well you’re not here to talk about your partner. Talk about your medication. But they don’t see past that.

Thus, in addition to project staff seeking to build trust with participants and convey trust for participants’ decisions, intervention activities grounded in the IIT-ICM commonly fostered participants’ own trust in and honesty with themselves. This honesty with oneself was described by a number of participants as a critical aspect of their behavior change decisions and processes. We examine the role of a related concept, self-reflection, in more detail in the theme described below.

### Opportunities for self-reflection on a range of topics and its effects

Aspects of the IIT-ICM, including non-judgment, trust building, compassion, and focus on the whole person were intended, in part, to foster open and honest self-reflection. Indeed, participants commonly reported significant, meaningful self-reflection during their time in the trial. In contrast, participants generally did not recall being asked about aspects of their lives unrelated to HIV in most other social service and health care settings. This, in turn, created a sense in those environments that their multilayered lived experiences should be kept separate from their HIV care management. Moreover, while the IIT-ICM assumed that emotions are an important aspect of HIV management, participants commonly stated they had never been asked about their emotional relationships with HIV medication or their HIV care in these other settings. Participants noted that reflecting on these fundamental questions about the experience of living with HIV allowed them to hear some of their own thoughts out loud for the first time, giving them the opportunity to notice patterns of behaviors in their lives. For some, these new insights about patterns of behavior and their causes precipitated behavior changes in HIV medication use, HIV care, mental health support seeking behavior, and substance use management.

In some cases, self-reflection, an intended outgrowth of the IIT-ICM, was directly linked to a new awareness of the importance of autonomy and self-determination in health care decisions. Samuel, a Black, gay, cisgender male in their late 30 s, who had been living with HIV for nearly 20 years, said they experienced a major shift in the way they looked at their life during the trial:

The counselor [at Heart to Heart 2], she's a real sweetheart. She never judged me, and never made a decision for me. She just asked me, "Well, how do you feel that decision has affected you?" And I never really thought about that. It was just a life-changing moment. Something just clicked. Like, yeah, that [the decision] is still not helping me. So, I want to do everything I can to help me, even though I still have to go through these dilemmas. I'm going to make sure that I'm all right.

Further, non-judgement may be essential for self-reflection in this context. Indeed, the importance of non-judgment was present in each of the themes described in this section.

Emmanuel was a Latino person assigned male sex at birth in their early 40 s who identified as bisexual and gender-fluid (that is, their gender identity did not fit inside traditional male or female categories). Emmanuel had lived with HIV for approximately 10 years, and had struggled for most of those years with HIV care engagement and HIV medication adherence. They explained the complex nature of their difficulties remaining in HIV care prior to joining the trial, which included difficulties accepting their HIV status, feeling judged by multiple providers due to their sexual orientation and gender identity, a perceived lack of confidentiality in some service provider settings, and extensive substance use over the years. As they reflected on their time in the trial, they said they experienced, “…a warm welcome when I came. You want some coffee? You want some tea? I was like, oh my God, that's nice. I wasn't [expecting that].” These elements of the trial were intended to foster relatedness, one aspect of the IIT-ICM. They went on to discuss their experience of being asked questions “they had never been asked before” regarding what they thought was best for them, recalling that, “they asked me what I wanted to do with my life”. Emmanuel said the project environment and the direct questions asked in the intervention components, both of which communicated caring and acceptance to them, opened up space for them to reflect on their life and health:

It makes you think about your life…. The generosity, the caring-ness you know? Somebody that doesn't even know you – they care about you, and to listen to you – what do you want? What do you want to do? You don't get asked them [usual] questions. People judge. People assume [they] already [know] what you want instead of asking you what you want. They're judging for you, or they're making a decision for you, and I don't want nobody to make a decision for me. Ask me what I want? Then you can point me to – if you have another option that we can work around or work with, then I have no problem with that. I have no problem with listening to what you have to say… I don't ever feel pressure here. No, I feel love here.

For Emmanuel, therefore, positive relationships with the project and project staff and non-judgment were preconditions for engagement in behavioral intervention components that, in turn, spurred self-reflection. Further, self-determination, a core element of the IIT-ICM, was clearly a deeply held value for Emmanuel.

William, a Black, heterosexual, cisgender male in their early 50 s, who had been living with HIV for 20 years, discussed developing a pervasive substance use “addiction” at an early age, which led them to leave home and to become estranged from their family for more than 30 years. During that time, and due largely to their substance use challenges, William was diagnosed with HIV and struggled to remain in HIV care and on HIV medication. Further, they subsequently experienced homelessness for several years, but, more recently, were stably housed with a family member they had reconnected with. William looked back on their time before starting the trial and reflected on the changes they had experienced in their life since starting the trial, related in large measure to self-reflection that took place during trial activities:

But this place has done a lot for me, looking at me, looking at the things that I’m doing, the things I’ve been going through, and all that other mess. You kept me thinking about what’s going on with me. You kept me thinking about taking my medication. Before I had gotten here to this place, I was on a sketchy – I may – I get my medication, and I sell it. Okay. I may sell like three months in a row. And I ain’t have no medication for the three months and didn’t care. But when I turned around and came here, and then we started talking, and I started listening to what I was saying, that’s the thing that’s helped me a lot. Listening to what the words that were coming out of my mouth. And I did not like it. I was self-destruct[ing], selling my meds, smoking crack, smoking so much crack that I was about ready to die. I didn’t care. And through coming through here and listening to myself speak and telling you all everything that’s been going on, and then how I’ve been doing with my medication and all this other mess, was making me look at – damn, do you want to die? Damn. You up here now with your [family]. Don’t you want to see them graduate from school, high school, going on to college? I was like, yeah, I do. I really do. I’d like to see them grow up and get married. I’m only [age redacted]. I still got some time left here on this earth. As long as I take my medication. You know. As long as I take my medication, I’m good.

Thus, for William, the research project environment and specific components grounded in the IIT-ICM fostered open and honest self-reflection, and this experience of “listening to […] the words that were coming out of my mouth” had a profound impact on their intentions regarding substance use patterns and HIV medication. Similarly, Wallace, introduced above, noted feeling committed to helping people who are suffering, but that they sometimes got in the way of “being there for myself”. They described experiences in the trial as a time for them to “to sit down and talk about these things out loud so that you can actually get it, process it,” and “I started listening to what I was saying.” These clinical experiences helped them to understand their own decisions and behaviors in a new and different way, which appeared to foster Wallace’s more serious engagement in the behavior change process, rather than passive participation. This, thereby, helped them re-evaluate their behavior patterns (heavy substance use, selling HIV medication) and make changes:

It’s one thing to just think things or hear someone else talk to you about it. But when you have a conversation about it where you’re involved in the conversation or a part of the conversation, it makes a difference. You process things a little differently, and it becomes more important to you.

Further, Wallace’s quote above underscores the importance of the IIT-ICM in eliciting discussion of behaviors such as heavy substance use, and selling/diverting HIV medications. These behaviors are not generally considered socially acceptable, but candid acknowledgement of and self-reflection about them are vital aspects of the behavior change process for many.

### Support of personal autonomy and its effects on motivation and decisions

As described above, participants generally experienced a lack of support for their personal autonomy in health care settings, particularly with regard to HIV care and decisions related to HIV medication. In particular, participants recalled feeling unable to advocate for themselves effectively in primary medical care and other social services environments. Although participants valued their individual providers, for the most part, HIV primary care settings were described as locations where they did not feel sufficiently valued or listened to, and participants discussed feeling “stuck” and unsure if there were other options available to them; for example, regarding where else they might be able to receive care. In contrast, consistent with the IIT-ICM, participants noted the development of a sense of personal autonomy throughout their time in the optimization trial. Some recalled feeling unexpectedly empowered to change medical providers if they felt they were not receiving the care they needed and deserved. They also expressed renewed feelings of autonomy regarding their ability to take HIV medication regularly, remain in HIV care, and reduce substance use or substance use-related harms. Samuel, introduced above, recalled that prior to starting in the trial they were feeling discouraged by the bureaucratic nature of the health care system, particularly at the primary care clinic they attended. They noted that experiences with the clinic, such as frustrations making appointments, made it extremely difficult to maintain their HIV care, leading them to stop caring about their health, which in turn contributed greatly to their stopping their HIV medication regimen. Samuel then described the changes they experienced in navigating their health care and HIV medication use after participating in the trial for a year:

Today, those same dilemmas are still there. Like, none of them have changed, but I've definitely changed a lot. You know what I'm saying? In my view of things, how I take care of myself, and how important it is to me to be medicated and stay healthy – and really almost taking my power back, my life, my control. So, it was really a big whirlwind. Even now thinking about this, like wow, honestly and truthfully somebody saved my life for $15 [the compensation the peer received for referring him to the study].

When asked how the project helped with aspects of HIV and other health management, Samuel described the following:

Okay, well basically the whole program to me – they were never judgmental, and they never made a decision for me. They just informed me of where I was and what decisions – and began to help me put in perspective what decisions were most important to me, and how in that plan I would respond differently when faced with different dilemmas.

Samuel’s experience, therefore, provides support for the importance of non-judgment and personal decision making, both of which are aspects of the IIT-ICM. Olivia, a Latinx, gay, transgender female approaching the age of 50 years, who had been living with HIV since they were a teenager, explained that they go “back and forth” with taking HIV medication regularly, since “I get tired of taking them after a while”. Olivia did not start HIV medication until they were in their early 40’s. They attributed this decades-long period of not taking HIV medication to negative experiences they had in the hospital when they were first diagnosed with HIV in the early days of the epidemic, along with a general disdain for taking pills and feeling like HIV medication was a constant emotional reminder of their HIV status. In reflecting on their experiences in the trial, Olivia said they felt more inclined to decide to take HIV medications as a result of their time in the trial:

Because you get information from each other here and, you know, sometimes when you do better things, when you go to better places and you do better things, it makes you feel better and you want to do better. So you know what I'm saying? […] Because you do better. Once you do better, you feel better about yourself, and that makes you want to do better. It's just like – simple. People just make it hard. Make it hard for ourselves, actually.

Marcus was a Black, gay, cisgender male in their early 50 s who was diagnosed with HIV when they were a teenager. Marcus noted “the majority of people that come [to the project], they come here from SROs [single-room occupancy residences], from drug usage, from prostitution, from the whole low income, promiscuous lifestyle.” As a result, they suggested, participants like themselves were generally reticent to speak openly with health care and social service providers about some of the more heavily stigmatized challenges they face. Marcus described their experience of being in medical environments where they felt like, “another piece of cattle just going through the chute.” They described this feeling as pervasive and added that it led them to stop attending appointments with multiple providers over the years. Marcus also discussed their substance use experiences extensively and also their challenges discussing substance use with providers:

I don't talk to them [providers] about substance use. And the reason why I don't is because I believe that relapse is a part of almost everyone's story, and a doctor that tries to insert their will would be a problem for me. Because there are doctors that'll say, "Okay, you have a substance abuse problem, okay, I'm going to put all your meds in a pharmacy bottle" [so he could not sell the medications]. That's a problem because that's not me keeping *myself* clean. That's you trying to force me to be clean. But they'll do it. So, and that, you're not gonna make decisions for me like that in my life. A doctor that is a 100 percent abstinence doctor, I don't need them. Not saying that I do not need abstinence because I do. But that's my choice. You're not gonna take that from me. So, me being articulate and intelligent allows me to see shit that a lot of other people will take for granted. And I can hear your words, and I understand what they mean, or I understand what you're telling me by not telling me. So, I pay attention.

Marcus, therefore, highlighted the importance of autonomy support and harm reduction, both aspects of the IIT-ICM, noting that a “100 percent abstinence” model is not useful to them. Similarly, Simone, introduced above, described their time in the project as valuable. Simone had a history of very difficult experiences with medical providers who Simone described as pressuring them to engage in certain health behaviors but not truly understanding their larger context or needs. According to Simone, during the trial they were able to traverse through a frustrating bureaucracy and finally find a clinic where they felt comfortable and could develop a good relationship with their provider:

I mean [I was] just facing issues that you don't normally want to face. You know, things that we keep on the inside that we do have issue with going to doctors’ appointments and we don't put the effort in, because of our own bias towards it. […] But if you could just get past that, there is help. But it's a mental thing. If you have one bad experience [in HIV care], you're kind of resistive on going back again or doubtful that they're going to help you. But I don’t feel like you should stop [trying]. There are hundreds of places you can go. Like just, all right, this one didn't work. Let me go here. This one didn't work. Okay, let me go here. And look, it only took me three times. Only three…. You know, so I pushed past my resistance and look, I fell into the perfect people [at an HIV care setting] for me.

In many instances, participants emphasized that while participating in the trial was useful, it was they themselves who carried the burden of managing HIV and they who must make the decision to take HIV medication, underscoring the importance of self-determination theory and autonomy support in the IIT-ICM. As Hank, identified above described,

I just take medication. And then here comes an oxymoron. My decision to take medication had nothing to do with you all and everything to do with you all. […] You made me think about it. And therefore, I started taking it. But it wasn't because you all forced me to do it or anything like that. It was just you put the thought in my head. People have been doing it around me for a little while. I just needed to hear from somebody else, because I was going to go the whole holistic thing. […] You know, herbs and all of that aromatherapy, but that shit doesn't work. Medication does.

Similarly, Samuel, described above, highlighted the utility of the IIT-ICM to guide participants toward behavior change, while supporting autonomy. Further, consistent with the IIT-ICM, which emphasizes evidence-based behavior change techniques including motivational interviewing, autonomy support alone is not generally sufficient for behavior change. Samuel said:

I was never pressured here to take medication. I was given options and choices, and discussed consequences. You know what I’m saying? Cause and effect, what this would – how this would affect me, you know what I’m saying? But never really given, you know what I’m saying, an ultimatum or this is what you need to do. You know what I’m saying? It was always all my decision, I felt. […] I was just given the tools to make that good decision.

## Discussion

The present qualitative and exploratory study focuses on a population of AABL-PLWH with socioeconomic disadvantage that experiences considerable barriers to HIV care and medication adherence, and, as a result, is either commonly not engaged, generally poorly engaged, or only intermittently engaged along the HIV care continuum. In response to the confluence of risk factors that impede HIV management in this population, and in the interest of ultimately creating new highly acceptable, feasible, and effective behavioral interventions to support sustained HIV viral suppression among AABL-PLWH, including those consistently poorly engaged along the HIV care continuum, our research team developed the IIT-ICM, which served as the foundation for the development of behavioral intervention components tested in an intervention optimization trial. Overall, results from the present study provide evidence for the IIT-ICM’s important role in the acceptability and perceived utility of the intervention components, and also in fostering engagement and therefore, in supporting study feasibility. Participants also reported a range of positive effects on behavior in response to the behavioral intervention activities grounded in the IIT-ICM. In particular, the present study’s results highlight how the IIT-ICM contributes to productive therapeutic and clinical processes between participants and interventionists, which, in turn, has potential to lead to improvements in quality of life and/or to behavior change.

### Trust and trustworthiness, power and privilege

AABL-PLWH are often caught in cycles of distrust and partial self-disclosure with their health care and social service providers, in part related to pre-existing medical distrust and counter-narratives, which most care settings do not address or are not well-equipped to address [15]. Further, relationships between AABL-PLWH and providers are shaped by treatment perspectives that prioritize total abstinence from substance use, and similarly, the expectation of taking HIV medication “every dose, every day.” Indeed, although harm reduction has gained some traction in HIV clinical settings, the abstinence-based/zero tolerance perspective, applied to substance use and HIV management, has deep, historical roots [26]. Yet, this zero tolerance/all-or-nothing approach in health care settings certainly impedes engagement along the HIV care continuum. Moreover, trust between medical or social service professional and AABL-PWLH is not easily established [84, 85]. But trust is vital to clinical and therapeutic interactions and self-change processes and fosters openness and self-reflection, but many research and clinical settings create environments and provide services that contribute to or perpetuate distrust [86–88]. Findings from the present study suggest that aspects of the behavioral intervention activities carried out in the present study and grounded in the IIT-ICM increased participants’ trust in the optimization trial and study staff in many cases, although overall medical distrust and counter-narratives may have persisted. Indeed, the goal of the trial was not to eliminate distrust or “correct” counter-narratives. In past research we found that AABL-PLWH can certainly engage in HIV care and services even while experiencing medical distrust and holding counter-narratives [15, 35], but that there is utility in eliciting and exploring these beliefs in the clinical context.

As shown in Fig. 2, the IIT-ICM highlights the need for cultural and structural salience and signals the need for intervention components to communicate cultural and structural competence, along with non-judgment and the support of autonomy, which in turn, are intended to foster trust between AABL-PLWH participants and the research project and its staff. Generally, we found participants in the present study engaged in frank discussions of their health decisions (e.g., not taking HIV medication) and contextual challenges (e.g., selling HIV medication, substance use) with project staff. These discussions included participants making the decision to discuss with project staff those behaviors not typically considered socially desirable or socially acceptable. Participants contrasted their experiences in the optimization trial with typical health care encounters. For example, substance use and substance use problems, both past and present, are very common among AABL-PLWH, but challenging to discuss in typical health care encounters, in part due to stigma and the short interval of time patients have with providers [27]. The optimization trial activities grounded in the IIT-ICM, with its emphasis on non-judgement and including harm reduction, and training of project staff in the IIT-ICM, may have played a role in this contrast between the optimization trial and typical care settings.

Participants in the present study were from very low socio-economic status contexts, while the research study was located in a well-funded academic institution and study staff were, by and large, more privileged than participants. In such cases, power imbalances are marked and can serve as impediments to trusting relationships [89]. We speculate that the IIT-ICM approach helped project staff and participants work collaboratively across these marked differences, since the IIT-ICM explicitly recognizes participants as the ultimate experts on their health decisions. Indeed, themes related to the importance of non-judgment from project staff, a lack of pressure to take HIV medication or achieve any other specific outcome defined by the study, and the important role that open and honest communication had on self-reflection (which often spurred behavior change) were prominent in the analysis.

### Health care settings are heavily influenced by the larger structural and policy context

The public health system and the individual HIV care providers within that system are charged with providing an effective and highly tolerable lifesaving medical treatment to individuals with a life-threatening condition, with the knowledge that such treatment can improve their wellbeing, provide them with a normal lifespan, and effectively eliminate the chances of their passing HIV onto others [5]. Study findings suggest current medical and social service settings may not be adequately prepared to address the confluence of serious barriers that some AABL-PLWH experience to HIV care continuum engagement. Indeed, there may be frustration on both sides: AABL-PLWH commonly express dissatisfaction with the ways they are viewed and the treatment and care they receive, including feeling pressured to take HIV medication, and HIV care settings and providers may be frustrated when patients do not take or adhere to HIV medication regimens [90]. Yet these service setting characteristics serve as direct and indirect barriers to engagement along the HIV care continuum: AABL-PLWH may avoid service settings they experience as insufficiently supportive of autonomy and that do not provide dignity-enhancing care, and, as we found in past research, these stresses, strains, insults, stigmas, and hassles are commonly internalized by AABL-PLWH over time, with grave adverse effects on one’s sense of self-worth, motivation to stay healthy, and HIV management behaviors [14]. Thus, the present study highlights the need for structural changes in order to end the HIV epidemic, along with enhancements to HIV care settings consistent with the IIT-ICM, as we discuss in more detail below.

### Autonomy support and motivational interviewing in health care settings

Although there are exceptions, in the present study HIV care and social service settings are generally described by participants as dismissive of issues that AABL-PLWH commonly consider relevant to HIV management, such as poor housing quality and relationship instability, but that providers may not see as directly related to engagement along the HIV care continuum. Further, AABL-PLWH are involved in numerous institutional systems and settings that surveil them and restrict their autonomy. Thus, AABL-PLWH find individualized, autonomy-supportive, and non-judgmental approaches largely lacking in the care and service settings in which they are engaged, although they tend to be satisfied with their individual health care providers [91]. As described above, grounded in the IIT-ICM, in the present study behavioral intervention activities were guided by the motivational interviewing approach [55]. There has recently been a modest emerging literature on the use of motivational interviewing among HIV care providers. For example, Flickinger and colleagues (2013) studied motivational interviewing-consistent behavior among untrained HIV care providers. They found untrained HIV providers do not consistently use motivational interviewing techniques when counseling patients about sexual risk reduction. However, when they do, their patients are more likely to express intentions to reduce sexual risk behavior [92]. Beach and colleagues (2018) trained HIV clinicians in motivational interviewing and found improvements in overall communication measures and patient experiences [93]. Laws and colleagues (2015) caution that routine health care encounters are typically too brief for client talk to evolve toward change and health care providers are not generally trained as mental health counselors [94]. Providers with limited training may have particular difficulty maintaining motivational interviewing consistency with resistant clients.

### Cultural and structural salience of components

The resonance of harm reduction and self-determination theory, core elements of the IIT-ICM, were prominent in the analysis. The role of critical race theory in the IIT-ICM were not as apparent in this analysis as aspects of the other theories or approaches, but because critical race theory was integral to the type, structure, and content of the study activities and intervention content (e.g., the role of poverty and housing, signaling the need for structural and cultural competence and salience, centering the project on AABL-PWLH), we do not consider it any less important. Indeed, throughout the project activities, participants were made aware that the optimization trial was focused on and interested in issues that shape the lives of AABL-PLWH such as structural racism, medical distrust, and counter-narratives about the causes and treatments of HIV. Thus, we assume that we cannot interpret findings such as the resonance of the autonomy supportive and non-judgmental approaches or harm reduction without acknowledging the contribution of critical race theory to the IIT-ICM.

### Limitations

The study has limitations including the possible influence of social desirability bias on findings. We sought to minimize social desirability bias during the interview process by asking general questions first and reminding participants they could and should feel free to decline to answer any question without penalty. The primary qualitative interviewer was not someone participants had previously met or worked with, as a further means of reducing social desirability bias. The present study does not evaluate the effectiveness of the intervention components or seek to optimize the intervention, which will be the focus of future research. Instead, we explored participants’ overall experiences with the optimization trial. The acceptability, feasibility, and potential impact of the specific intervention components tested in the optimization trial will be presented in a subsequent study, along with ways the components could be improved from the perspectives of participants. The behavioral intervention components grounded in the IIT-ICM appear highly acceptable, but the optimization trial may have been better resourced than many HIV care settings. Indeed, the components were designed to be easy to access, flexible, and easy to navigate, but not all service settings have the resources to take such an approach. Indeed, there are disparities in funding for HIV care and social service settings, along with structural barriers such as long travel times to clinics in locations with high-HIV burdens [95, 96]. The Health Resources and Services Administration’s Ryan White HIV/AIDS Program (RWHAP) has made significant progress reducing disparities in HIV care continuum engagement among populations at risk, including women, African American and Black PLWH, and unstably housed clients [97]. Yet, as described throughout the present study, gaps remain.

### Implications

In Table 3 we present a number of implications for policy, health departments, and HIV care delivery models drawn from the present study. These implications fall into two main categories: implications for the larger contexts in which AABL-PLWH are located, including structural/systemic changes needed, and implications for HIV care settings. The present study indicates it will be necessary to simultaneously address structural barriers and improve HIV care delivery models to engage this subpopulation of AABL-PLWH along the care continuum consistently. For example, chronic poverty is a core cause of disengagement along the HIV care continuum [98], along with poor-quality housing [99]. The present study provides insights into potential alternate approaches that can be carried out within settings. Co-located substance use and HIV services, funding for high-quality supportive housing, and collaborative patient-provider relationships could improve sustained viral suppression among populations experiencing constellations of challenges such as substance use, poverty, and long-term HIV [100]. The present study also suggests there would be utility in some cases for a comprehensive, top-to-bottom design or re-design of HIV care delivery models, HIV care settings, and social service settings for AABL-PLWH guided by the IIT-ICM or similar model, including involving AABL-PLWH in the planning process. Fox and colleagues (2014a, 2014b) describe such a model; namely, an urban transitions clinic serving formerly incarcerated persons that evidences promising engagement and health outcomes. Yet, similar to the present study, Fox and colleagues note that that access to medical care is necessary but not sufficient to control chronic health conditions for this population with structural barriers to heath [101, 102]. In Table 4 we provide practical guidelines for designing behavioral interventions/intervention components using the IIT-ICM.

**Table 3:** Implications drawn from the present study

Implications for the larger context in which AABL-PLWH are located
** It will be necessary to address structural inequality to end the HIV epidemic**. Structural racism and structural inequality are fundamental causes of poor engagement along the HIV care continuum, and it is possible to address structural factors. For example, poverty is a fundamental cause of poor engagement and could be eliminated through poverty-reduction measures such as universal basic income or increasing federal financial benefit levels
** In addition to addressing structural inequality, it will be necessary to tailor HIV care delivery models to the needs of AABL-PLWH to end the HIV epidemic**. To bring this subpopulation of AABL-PLWH onto the HIV care continuum consistently, it will be necessary to simultaneously address structural barriers to engagement, and enhance or modify HIV care delivery models
** High-quality housing is a foundation of HIV management**. In some geographical locations, housing support for PLWH is needed, but lacking. In other more service-rich settings, such as New York City, housing support is provided, but poor-quality housing such as single-room occupancy residences interferes with HIV care continuum engagement and wellbeing generally
** Data science has a role to play in ending the HIV epidemic**. AABL-PLWH tend to discontinue HIV medication when life circumstances change. Data science may play a role in efforts to predict and prevent HIV medication discontinuation along with resources (adequate financial benefits, high-quality housing) to buffer the effects of life changes
** Corrupt pharmacies undermine efforts to end the HIV epidemic**. Ongoing efforts to stop pharmacies from illegally buying HIV medication from patients are needed
Implications for HIV care delivery settings
** Structural competence may be lacking in many care settings**. HIV care settings can be designed or re-designed in a comprehensive, top-to-bottom approach guided by models such as the IIT-ICM and by involving AABL-PLWH in the design process
** Collaborative care approaches may be lacking in many settings**. Health care providers can be trained in motivational interviewing, harm reduction, and other collaborative approaches as part of a comprehensive approach to addressing the problem of poor engagement along the HIV care continuum
** Health care encounters are short but barriers to engagement are serious**. Since health care encounters tend to be short, health care providers can better partner with social service providers and behavioral interventionists to meet the needs to those with the greatest barriers to engagement along the HIV care continuum
** PLWH not taking HIV medication often feel unwelcome in HIV care**. Not all AABL-PLWH are ready to take HIV medication at any given time and those not taking medication often leave or are even pushed out of HIV primary care. Enhanced efforts to locate and engage AABL-PLWH not taking HIV medication in HIV primary care and other services are warranted
** Substance use and mental health treatment in HIV care settings may not be sufficiently available and/or may benefit from the IIT-ICM**. Substance use and substance use challenges and mental health concerns are very common among AABL-PLWH who are poorly engaged along the HIV care continuum, but harm reduction and dignity-enhancing services are lacking. Co-locating HIV care, substance use (including harm reduction approaches), mental health, navigation, and other needed services will boost engagement in these services
** Care settings may not address emotions inherent in HIV management sufficiently**. Many programs and interventions for AABL-PLWH do not sufficiently attend to the emotions inherent in HIV management, but the IIT-ICM and the present study underscore the important role of emotion in engagement along the HIV care continuum,
** Patients and providers often have fraught relationships**. Patients’ relationships with providers are often complicated by fear and medical distrust, the tendency to give socially desirable responses, and experiences of stigma, in the context of short health care encounter times. Approaches grounded in the IIT-ICM can foster more constructive and open communication and relationships
** Power imbalances are common between AABL-PLWH from socioeconomically disadvantaged backgrounds and providers, which may impede open and honest communication.** Counseling and treatment approaches grounded in the IIT-ICM can potentially play a role in fostering open communication and trust across these imbalances
** Individualized and flexible approaches are needed**. Meeting AABL-PLWH “where they are” by addressing the health needs patients prioritize first can generate trust and foster the needed constructive provider/participant relationship, which has the potential to generate additional health goals
** Disengagement from the care continuum can be prevented**. Retention clinics within HIV care settings can provide targeted services to AABL-PLWH at risk for disengagement

**Table 4:** Practical guidelines for designing behavioral interventions using the IIT-ICM

General principles	•Involve members of the population under study in all steps of this analysis and design process (e.g., participatory action research models) •Involve content experts in both upstream (systemic/distal) and downstream (proximal) factors in all steps of this analysis and design process, including those with lived experience •Use these steps in an iterative manner and return to previous steps to revise the analyses, model, and intervention content as needed
Step 1	Identify the public health problem to address
Step 2	Define the specific behavior to change (the behavior of interest)
Step 3	Identify the **upstream historical, structural, and systemic factors** that create or contribute to this public health problem and analyze how they influence the behavior of interest •“Center the margins” to prioritize the perspectives of the population under study, rather than the dominant group •Consider racism and inequality from a systemic lens Elicit and understand counter-narratives that may influence behavior change •Consider how systems and structures intersect to create risk (called “structural intersectionality”) •Identify sources of population- and individual-level resistance, strengths, and resilience
Step 4	Identify the more **proximal downstream factors** that promote or impede the behavior of interest (e.g., attitudes, beliefs, social factors, social network factors, access factors)
Step 5	The range of factors that influence the behavior of interest, both upstream and downstream and modifiable and non-modifiable, have now been identified. These modifiable factors can now be organized into a conceptual model using **the theory of triadic influence** (or a similar flexible multi-level theory) that articulates multiple levels of influence on behavior; e.g., structural-, social-, and individual/attitudinal levels of influence •Not all upstream and downstream factors will need to be placed in the resultant conceptual model, but the model should reflect the primary potentially modifiable factors that promote/impede the behavior of interest •Ideally, the factors in the model will be addressed in the intervention/intervention components and be conceptualized as mediators of the intervention
Step 6	To develop the specific intervention or intervention components and the optimal behavior change techniques, at this step bring in an **existing intervention development framework** such as intervention mapping (Bartholomew) or the behavior change wheel (Michie) •The intervention or intervention components will entail specific **behavior change techniques.** A behavior change technique is an observable and replicable component designed to change behavior (e.g., habit formation, problem solving, social support, self-talk, review of behavioral goals) •Consider how the behavior change techniques relate to the IIT-ICM •Some behavior change techniques will align better with the IIT-ICM than others •Interventions/intervention components generally also have a **counseling or delivery method or approach,** which are guided by theory and may entail of multiple behavior change techniques. Examples include cognitive-behavioral therapy, mindfulness-based therapy, behavioral therapy, group counseling, and motivational interviewing •Motivational interviewing aligns with the IIT-ICM. Consider whether the motivational interviewing approach would enhance the intervention/intervention components
Step 7	Evaluate how the intervention structure, modalities, delivery, and content will be implicitly and explicitly **structurally salient** •Ask whether the intervention implicitly and explicitly locates the primary causes of the public health problem at an upstream level and evaluate whether this is communicated in the intervention content •E.g., interventions can guide participants through an analysis of barriers to a health problem that starts with structural/systemic causes
Step 8	Evaluate how the intervention structure, modalities, delivery, and content will be implicitly and explicitly **culturally salient** •Examples include introducing and/or listening for culturally salient factors such as medical distrust, fear of medications, counter-narratives about health problems, and attending to sources of resistance and resilience grounded in culture
Step 9	Evaluate the intervention or intervention components for the following characteristics and revise as needed. How are they implicitly and explicitly **supportive of personal autonomy, trust building, de-stigmatizing, non-judgmental, and dignity enhancing**? Are there **no pre-conditions** for treatment? Do intervention components guide toward **any positive change**?

## Conclusions

Study findings suggest that the behavioral intervention activities grounded in the IIT-ICM and delivered as part of the optimization trial were experienced by participants as highly acceptable, relevant, culturally and structurally salient, and distinct from clinical activities and social services received in other settings. Further, findings suggest the grounding of the study activities in the IIT-ICM contributed to participants’ engagement in the optimization trial, and, therefore, to high rates of retention and study feasibility. Results also suggest that the intervention components yielded useful effects on HIV management in some cases, along with effects on general wellbeing and quality of life, as well as on idiosyncratic outcomes. Thus, as we hypothesized at the time the IIT-ICM was created, the three theories/approaches that comprise the IIT-ICM, namely, critical race theory, harm reduction, and self-determination theory, appear to combine synergistically to create a new and useful model. The IIT-ICM may have applications for policy, HIV care delivery models, and other AABL populations in high-risk contexts, and warrants further study. The present study explored participants’ experiences in the optimization trial as a whole. In a future study we will explore in more detail the acceptability, feasibility, and impact of the six specific intervention components tested in the optimization trial and ways they can be improved from the participants’ perspectives.

## Supplementary Information

**Additional file 1.**

## Data Availability

Data are available upon reasonable request from the corresponding author.

## Abbreviations

AABL: African American/Black and Latino
CDC: Centers for Disease Control and Prevention
HIV: Human immunodeficiency virus
IIT-ICM: Intervention Innovations Team integrated conceptual model
PLWH: Persons living with HIV
PTSD: Post-traumatic stress disorder
REDCap: Research Electronic Data Capture
