# Correction: Advancing behavioral interventions for African American/Black and Latino persons living with HIV using a new conceptual model that integrates critical race theory, harm reduction, and self-determination theory: a qualitative exploratory study

**DOI:** 10.1186/s12939-022-01708-2

**Published:** 2022-08-17

**Authors:** Marya Gwadz, Sabrina R. Cluesman, Robert Freeman, Linda M. Collins, Caroline Dorsen, Robert L. Hawkins, Charles M. Cleland, Leo Wilton, Amanda S. Ritchie, Karen Torbjornsen, Noelle R. Leonard, Belkis Y. Martinez, Elizabeth Silverman, Khadija Israel, Alexandra Kutnick

**Affiliations:** 1 Intervention Innovations Team Lab (IIT-Lab), New York University Silver School of Social Work, New York, NY USA; 2 Center for Drug Use and HIV Research, School of Global Public Health, New York University, New York, NY USA; 3 Independent Consultant, Brooklyn, NY USA; 4 Department of Social and Behavioral Sciences, School of Global Public Health, New York University, New York, NY USA; 5 Rutgers University School of Nursing, Newark, NJ USA; 6 Department of Population Health, Division of Biostatistics, New York University School of Medicine, New York, NY USA; 7 Department of Human Development, State University of New York at Binghamton, Binghamton, NY USA; 8 Faculty of Humanities, University of Johannesburg, Johannesburg, South Africa; 9 School of Global Public Health, New York University, New York, NY USA; 10 Department of Psychology, Fordham University, Bronx, NY USA

**Correction: Int J Equity Health 21, 97 (2022)**

**https://doi.org/10.1186/s12939-022-01699-0**

After publication of this article [1], the authors reported that the caption to Fig. 2 (‘IIT-ICM core elements for ICM1’) should have read ‘Core elements and key characteristics of the Intervention Innovations Team integrated conceptual model (IIT-ICM) to guide behavioral intervention development’.

The original article [1] has been corrected.
